# Pharmacological Inhibition of the Spliceosome SF3b Complex by Pladienolide‐B Elicits Craniofacial Developmental Defects in Mouse and Zebrafish

**DOI:** 10.1002/bdr2.2404

**Published:** 2024-11-04

**Authors:** Yukiko Hoshino, Shujie Liu, Toshiko Furutera, Takahiko Yamada, Daisuke Koyabu, Yuko Nukada, Masaaki Miyazawa, Tetsuya Yoda, Koichiro Ichimura, Sachiko Iseki, Junichi Tasaki, Masaki Takechi

**Affiliations:** ^1^ Department of Molecular Craniofacial Embryology and Oral Histology, Graduate School of Medical and Dental Sciences Tokyo Medical and Dental University (TMDU) Tokyo Japan; ^2^ Office of Vaccines Pharmaceuticals and Medical Devices Agency (PMDA) Japan; ^3^ R&D, Safety Science Research, Kao Corporation Kawasaki Japan; ^4^ Department of Anatomy and Life Structure Juntendo University Graduate School of Medicine Tokyo Japan; ^5^ Department of Maxillofacial Surgery, Graduate School of Medical and Dental Sciences Tokyo Medical and Dental University (TMDU) Tokyo Japan; ^6^ Research and Development Center for Precision Medicine University of Tsukuba Ibaraki Japan; ^7^ R&D, Safety Science Research, Kao Corporation Tochigi Japan

**Keywords:** neural crest cell, neural tube defect, pladienolide‐B, RNA splicing, SF3b complex, spliceosomopathy

## Abstract

**Background:**

Mutations in genes encoding spliceosome components result in craniofacial structural defects in humans, referred to as spliceosomopathies. The SF3b complex is a crucial unit of the spliceosome, but model organisms generated through genetic modification of the complex do not perfectly mimic the phenotype of spliceosomopathies. Since the phenotypes are suggested to be determined by the extent of spliceosome dysfunction, an alternative experimental system that can seamlessly control SF3b function is needed.

**Methods:**

To establish another experimental system for model organisms elucidating relationship between spliceosome function and human diseases, we administered Pladienolide‐B (PB), a SF3b complex inhibitor, to mouse and zebrafish embryos and assessed resulting phenotypes.

**Results:**

PB‐treated mouse embryos exhibited neural tube defect and exencephaly, accompanied by apoptosis and reduced cell proliferation in the neural tube, but normal structure in the midface and jaw. PB administration to heterozygous knockout mice of *Sf3b4*, a gene coding for a SF3b component, influenced the formation of cranial neural crest cells (CNCCs). Despite challenges in continuous PB administration and a high death rate in mice, PB was stably administered to zebrafish embryos, resulting in prolonged survival. Brain, cranial nerve, retina, midface, and jaw development were affected, mimicking spliceosomopathy phenotypes. Additionally, alterations in cell proliferation, cell death, and migration of CNCCs were detected.

**Conclusions:**

We demonstrated that zebrafish treated with PB exhibited phenotypes similar to those observed in human spliceosomopathies. This experimental system may serve as a valuable research tool for understanding spliceosome function and human diseases.

## 1 Introduction

Splicing is a crucial regulatory process of gene expression that expands the repertoire of proteins produced from a single gene. The spliceosome is an RNA and protein complex essential for recognizing noncoding introns in precursor messenger RNA (pre‐mRNA) and processing splicing to connect exons. There are two types of splicing machineries, the major and minor spliceosome. The major spliceosome comprises small nuclear ribonucleoproteins (snRNPs), U1, U2, U4/U6, and U5, along with seven core proteins common to all snRNPs (Sm proteins), and a variable number of associated proteins (Griffin and Saint‐Jeannet 2020). Among these components, the U2 snRNP is an essential component in splicing process. It includes two protein complexes, Splicing Factor 3a (SF3a) and 3b (SF3b). The SF3a complex plays a crucial role in the formation of the active splicing site, while the SF3b complex is responsible for identifying the branch point sequence within the intron (Golas et al. 2003). The SF3b complex is comprised of seven subunits: SF3B1, SF3B2, SF3B3, SF3B4, SF3B5, SF3B14 (SF3B6), and PHF5A (SF3B7) (Sun 2020). Of these, SF3B1 is a central splicing protein that stabilizes the binding between the U2 snRNA and the branch point in the RNA target (Gozani, Feld, and Reed 1996; Gozani, Potashkin, and Reed 1998). SF3B4 binds to the pre‐mRNA in close proximity to the branch point sequence, ensuring sustained attachment of the U2 snRNP to the branch site (Champion‐Arnaud and Reed 1994).

The spliceosome constitutes an indispensable apparatus in all cell and tissue types, playing a vital role in the splicing process. Its components are ubiquitously expressed in embryonic development (An and Henion 2012; Devotta et al. 2016; Lei et al. 2017; Yamada et al. 2020). Despite this widespread expression, mutations in genes encoding spliceosome components often result in cell‐ or tissue‐specific abnormalities, impacting the development of specific organ systems such as the brain, retina, hematopoietic lineage, craniofacial skeleton, spinal cord, and limbs in humans. Such diseases include Burn‐McKeown syndrome and cerebrocostomandibular syndrome, collectively referred to as spliceosomophaties (Beauchamp et al. 2020; Griffin and Saint‐Jeannet 2020; Lehalle et al. 2015; Merkuri and Fish 2019). Mutations in components in the SF3b complex, such as *SF3B2*, *SF3B4*, and *PHF5A*, along with other several splicing factors, are associated with craniofacial defects such as micrognathia, microtia, and prearticular tags (Beauchamp et al. 2020; Harms et al. 2023; Lehalle et al. 2015; Timberlake et al. 2021).

Nager syndrome (NS) is predominantly caused by nonsense mutation in *SF3B4*, accounting for approximately 60% of NS cases (Bernier et al. 2012; Czeschik et al. 2013; Petit et al. 2014; Ulhaq et al. 2023a). NS manifests as acrofacial dysostosis, featuring malar and mandibular hypoplasia, downward slanted palpebral fissures, micrognathia, cleft palate, hearing loss, absent thumbs, and radial hypoplasia. Myelodysplastic syndromes (MDS) are hematopoietic disorders, characterized by abnormal hematopoiesis leading deficiencies in hematopoietic lineages (Catenacci and Schiller 2005; Fei et al. 2018). Splicing factors with mutations linked to MDS are predominantly associated with the U2 complex, including *SF3B1* (Griffin and Saint‐Jeannet 2020). These previous reports have indicated that the affected organs vary depending on which component of the SF3b complex undergoes mutations and at which position these mutations occur. This is partially because the conformational changes of the SF3b complex varies depending on the position of the mutations, and the degree of intron recognition and binding site accuracy of the SF3b complex varies in various way (Alsafadi et al. 2016). Consequently, the degree of SF3b complex dysfunction, including the types and numbers of pre‐mRNAs affected by splicing inhibition, varies among different spliceosomopathies (Marques et al. 2016; Dolatshad et al. 2016). In light of this context, an experimental system capable of seamlessly controlling the function of the SF3b complex and analyzing its impact could offer valuable insights into this kind of human diseases.

A number of model organisms have been generated through genetic modifications of spliceosome components (Olthof, White, and Kanadia 2022). Regarding the SF3b complex, homozygous deletion of *Sf3b1* or *Sf3b4* in mice leads to early embryonic lethality (Isono et al. 2005; Yamada et al. 2020), as observed with other splicing factors (Baumgartner et al. 2018; Beauchamp et al. 2021). Heterozygous deletion of these genes affects axial skeleton and brain development but not the craniofacial skeleton, failing to recapitulate human spliceosomopathy phenotypes (Isono et al. 2005; Kumar et al. 2023; Yamada et al. 2020). In contrast, studies employing morpholinos targeting *Sf3b2* and *Sf3b4* in *Xenopus* have demonstrated defects in neural crest and craniofacial development (Devotta et al. 2016; Timberlake et al. 2021). In zebrafish, the mutant with significantly reduced expression of *sf3b1* affects the development of neural crest derivatives including pigment cells and cranial nerves (An and Henion 2012). Homozygous deletion of zebrafish *sf3b4* leads to abnormal development of the retina and craniofacial skeleton (Ulhaq et al. 2023b; Ulhaq and Tse 2024).

Various small molecules disrupt the precise binding between the spliceosome and pre‐mRNA, resulting in the splicing inhibition (Effenberger, Urabe, and Jurica 2017). Of these, Pladienolide‐B (PB) and several compounds function as spliceosome modulators targeting the SF3b complex (Kotake et al. 2007; Yokoi et al. 2011; Carvalho et al. 2017). Previous in vitro studies have demonstrated that these chemicals exert their influence on the pocket formed by SF3B1 and PHF5A in the SF3b complex, competitively inhibiting the base pairing between the U2 snRNA and the pre‐mRNA branch point sequence (Teng et al. 2017; Finci et al. 2018; Larsen 2021). Consequently, splicing modulators affect splicing efficiency by disrupting splicing pattern (Teng et al. 2017). It has also been suggested that splicing efficiency decreases in a dose‐dependent manner with splicing modulators (Teng et al. 2017). We focus on the specificity of compounds that selectively inhibit the SF3b complex for in vivo study. By varying the dosage of the compound administered to embryos, it is possible to control splicing efficiency within a single analytical system. Therefore, in this study, to establish an alternative experimental system for knockout analysis or morpholino‐mediated deletion or reduction of expression of a SF3b component, we administer PB into mouse and zebrafish embryos and evaluate whether these embryos exhibit phenotypes resembling those observed in spliceosomopathies.

## 2 Materials and Methods

### 2.1 Mice

Two wild‐type mouse (*Mus musculus*) strains, C57BL/6J and ICR (Sankyo Labo Service), were housed in an environmentally controlled room at 23 ± 2°C, with a relative under a 12‐h light:12‐h dark cycle. Heterozygous knockout mouse of *Sf3b4* (*Sf3b4* ^*+/−*^, Yamada et al. 2020) was maintained on a C57BL/6J background. The morning on which a vaginal plug was found was defined as embryonic day (E) 0. All mouse experiments were conducted in Tokyo Medical and Dental University (TMDU) in accordance with protocols certified by the Institutional Animal Care and Use Committee of TMDU (A2019‐060C2). For genotyping for *Sf3b4* knockout mouse, genomic DNA was extracted from a part of the tissue of embryos. The primers which amplified the targeted region (5’‐GGCCCGGAAGTGGAAGTTGT‐3′ and 5’‐TGGGTTGAGCAGTGGCCTTT‐3′) were used for PCR. The PCR was performed by KOD FX polymerase (TOYOBO) with the following conditions: 1 cycle of 94°C for 2 min; 35 cycles of 98°C for 10 s and 68°C for 2.5 min. The PCR products were checked by electrophoresis with 1%–2% Tris‐Acetate‐EDTA (TAE) gels.

### 2.2 Zebrafish

The zebrafish (*Danio rerio*) strain RIKEN WT (RW) and *Tg*(*−5.0sox10:EGFP*) (RW background and referred as *sox10:EGFP*) were raised under a 14‐h light;10‐h dark cycle. The water temperature was carefully controlled within a range of at 28 ± 1°C and optimal water quality conditions were ensured by adhering to The Zebrafish Book (Westerfield 2000) and The Guide for the Care and Use of Laboratory Animals 8th edition (National Research Council 2011). The transgenic line *sox10:EGFP* was generated using Tol2 system, and maintained as heterozygotes or homozygotes (Liu et al. 2023).

### 2.3 Administration of Pladienolide‐B

For exposure of Pladienolide‐B (PB, Cayman Chemical, #16538) to mouse embryos by administering pregnant mouse, PB was dissolved in DMSO as 1 μg/μl solution and stored at −20°C. The solution of PB in corn oil at 300 μL/body for C57BL/6J and *Sf3b4* ^*+/−*^ mice while 350 μL/body for ICR mice was injected intraperitoneally. As for control mice, only corn oil was injected intraperitoneally. For administration of PB to zebrafish embryos, male and female zebrafish in the adult stage (4–10 months after fertilization) were placed in a breeding tank with a separator in the late afternoon of the day before spawning. The separator was removed in the morning and spawning was stimulated when the light was turned on. Fertilized eggs were collected within 1 h after removal of the separator. The eggs were incubated in E3 medium (5 mM NaCl, 0.17 mM KCl, 0.33 mM CaCl2, 0.33 mM MgSO4, adjust the pH to 7.2 with NaOH) at 28°C. At 4 h post‐fertilization (hpf), the eggs were exposed to PB. The exposure medium was replaced on a daily basis, and samples were collected starting from the 13 somite stage (ss) up until 120 hpf.

### 2.4 Transmission Electron Microscope (TEM)

Mouse embryos at E8.5 were fixed with 2.5% glutaraldehyde in 0.1 M phosphate buffer overnight. After washing in phosphate buffer, 1% osmium tetroxide in 0.1 M phosphate buffer was used for post‐fixation. These samples were processed by propylene oxide after dehydration. The propylene oxide was then replaced by epon resin and embedded in epon resin. Semi‐thin sections at 1 μm and toluidine blue staining were utilized for deciding the area for observing with TEM. Ultrathin sections were collected at 80 nm, and uranyl acetate and lead citrate on carbon‐coated copper grids were utilized for staining. The sections were observed with TEM (Hitachi, H‐7100).

### 2.5 Immunofluorescent Staining for Mouse Embryos

Mouse embryos were fixed with 4% paraformaldehyde (PFA) in phosphate buffered saline (PBS) overnight. These samples were processed by 5%–25% sucrose solution, embedded in Optimal Cutting Temperature (O.C.T.) compound (Sakura Finetek), and stored at –80°C until use for immunofluorescent staining. The samples were sliced into 10–12 μm frontal sections. The sections were washed by PBS and treated with 5% goat or horse serum for 30 min and then incubated with the primary antibody; N‐cadherin (BD Transduction, #610920, 1:200), Phospho‐Histone H3 (Cell Signaling Technology, #9701, 1:200), Cleaved Caspase‐3 (Cell Signaling Technology, #9661, 1:200), or AP2 (DSHB, #3B‐5, 1:100) overnight. These sections were then incubated with the secondary antibody conjugated with Alexa Fluor 488 or 555 (Invitrogen, 1:200) and Hoechst 33342 (Dojindo, 1:1000) for 30 min. The specimens were detected by BX53 microscope and DP80 camera system (Olympus) or DM68 microscope and DFC310 FX camera system (Leica Microsystems). For the detailed observation of N‐cadherin signals, a laser scanning confocal microscope (TCS SP8, Leica Microsystems) was used.

### 2.6 Histological Analysis for Mouse Fetuses

Mouse embryos at E13.5 were directly embedded in O.C.T. compound, fixed in Bouin's solution, or in Carnoy's solution (ethanol: acetic acid: formalin = 6:3:1). The whole‐mount samples were observed by SZX16 microscope and DP80 camera system (Olympus). The fixed samples were embedded in paraffin after dehydration, and frontal sections were obtained at 7 or 8 μm thickness. The sections were treated with 3% H_2_O_2_ in methanol for 20 min and performed antigen retrieval in sodium citrate buffer at 95°C for 20 min, followed by treated with 5% goat serum for 30 min. The sections were incubated with the primary antibody of RUNX2 (Cell Signaling Technology, #12556; 1:150) overnight and then incubated with the secondary antibody conjugated with biotin (Vector laboratories, BA‐1000; 1:200) for 30 min, followed by treatment in ABC solution (Vector laboratories, #PK‐6100, 1:100) for 30 min. Signals were visualized using DAB (TaKaRa #MK210). After immunostaining, the sections were stained with Alcian blue 8GX (Waldeck, 1A‐288, 0.1%) and Toluidine blue. For histological analysis of the retina, Hematoxylin–Eosin (Sakura Finetek) staining was performed. The slides were cleared by xylene and applied to mounting agent (Entellan new, Merck) for observation by BX53 microscope and DP80 camera system (Olympus).

### 2.7 Fluorescence Imaging and Immunofluorescence Staining for Zebrafish Embryos

Immunofluorescence staining of zebrafish embryos were carried out as previously described (Narumi et al. 2020; Liu et al. 2023). Briefly, the fixed earlier stage embryos (< 24 hpf) were permeabilized with 1% Tritonx‐100 (Cayman Chemical) in PBS more than 1 h, while 96 and 120 hpf samples were prepared followed by Narumi et al. 2020. After the blocking with 3% bovine serum albumin (BSA, Wako) in PBS‐T (PBS containing 0.1% Triton X‐100), embryos were incubated with the primary antibody of GFP (Millipore, MAB3580, 1:1000), Acetylated tubulin (Sigma‐Aldrich, T6793, 1:5000), Collagen type II (DSHB, AB_528165, 1:100), HuC/HuD (Thermo Fisher Scientific, 16A11, 1:100), Active caspase3 (BD Pharmingen, 559565, 1:1000), and Phospho‐Histone H3 (Ser10) (EMD Millipore, 06–570, 1:1000) or PNA lectin conjugated with Alexa Fluor 488 (1:1000) overnight at 4°C. The samples were washed six times with PBS‐T for 15 min and stained with the following secondary antibodies; Alexa Fluor 488 or 568 (Life Technologies, 1:1000), DAPI solution (Dojindo, 1:1000), and RedDot2 solution (Biotium, 1:200) overnight at 4°C. Following six times wash with PBS‐T for 15 min each, the samples were embedded in 1% low‐melting agarose (Sigma‐Aldrich) and mounted on a 27‐mm non‐coated glass bottom dish (Iwaki). For live imaging, the samples were anesthetized with 0.02% MS‐222 (Sigma‐Aldrich) and embedded in 1% low‐melting agarose (Sigma‐Aldrich) containing 0.02% MS‐222 on the glass bottom dish. All fluorescent staining samples and live samples were imaged on Zeiss LSM800 system equipped with Zeiss ZEN blue software and were presented as representative optical sections or maximum intensity projections. For 3D visualization, IMARIS software (Oxford instruments) was used. All procedures were performed at room temperature, unless otherwise mentioned.

### 2.8 Statistics

To compare the orientation of neuroepithelial cells, the angles of the long axes of the nuclei were measured for both control and PB‐treated embryos, and the mean angle for each individual was calculated. The difference between the angle of each cell and the mean angle was measured, and the degree of variance between the two groups was tested by *F*‐test using Microsoft Excel (Microsoft). *p*‐values for the ratio of the length and width in the neuroepithelial cell were calculated using Student's *t*‐test using GraphPad Prism (version 6, GraphPad Software). Positive cell rates of CC3 and PHH3 were calculated by the positive cell number per nucleus number in the same area. *p*‐values were calculated using Student's *t*‐test or one‐way ANOVA followed by Dunnett's multiple comparison tests using GraphPad Prism (version 6 or 8, GraphPad Software) for quantification of the number of PHH3‐ or CC3‐positive cranial neural crest cells (CNCCs). *p*‐values < 0.05 were considered statistically significant. All data are presented as the mean ± SD unless otherwise specified.

## 3 Results

### 3.1 Wild‐Type Mouse Embryos Exposed to PB Exhibited Neural Tube Defect

We first evaluated the conditions for PB exposure to the mouse embryo. We tested intraperitoneal PB administration to pregnant ICR mice at E8.5 with a dose of 0.6, 1.8, 3.6, and 7.2 mg/kg body weight (bw). In mothers administered with 0.6 mg/kg bw of PB, no specific behavioral alterations were observed, and no abnormal morphology was detected in embryos at E10.5. Conversely, mothers treated with 3.6 and 7.2 mg/kg bw of PB exhibited pronounced toxicity, resulting in inability to sustain pregnancy. Mothers administered with 1.8 mg/kg bw of PB did not display specific behavioral changes, but evident abnormal morphology were observed in embryos at E10.5. We also attempted to administer 1.8 mg/kg bw of PB continuously at 24‐h intervals but abandoned due to recurrent abortions. Thus, we decided to give a single‐dose administration of 1.8 mg/kg bw in this study.

**FIGURE 1:**
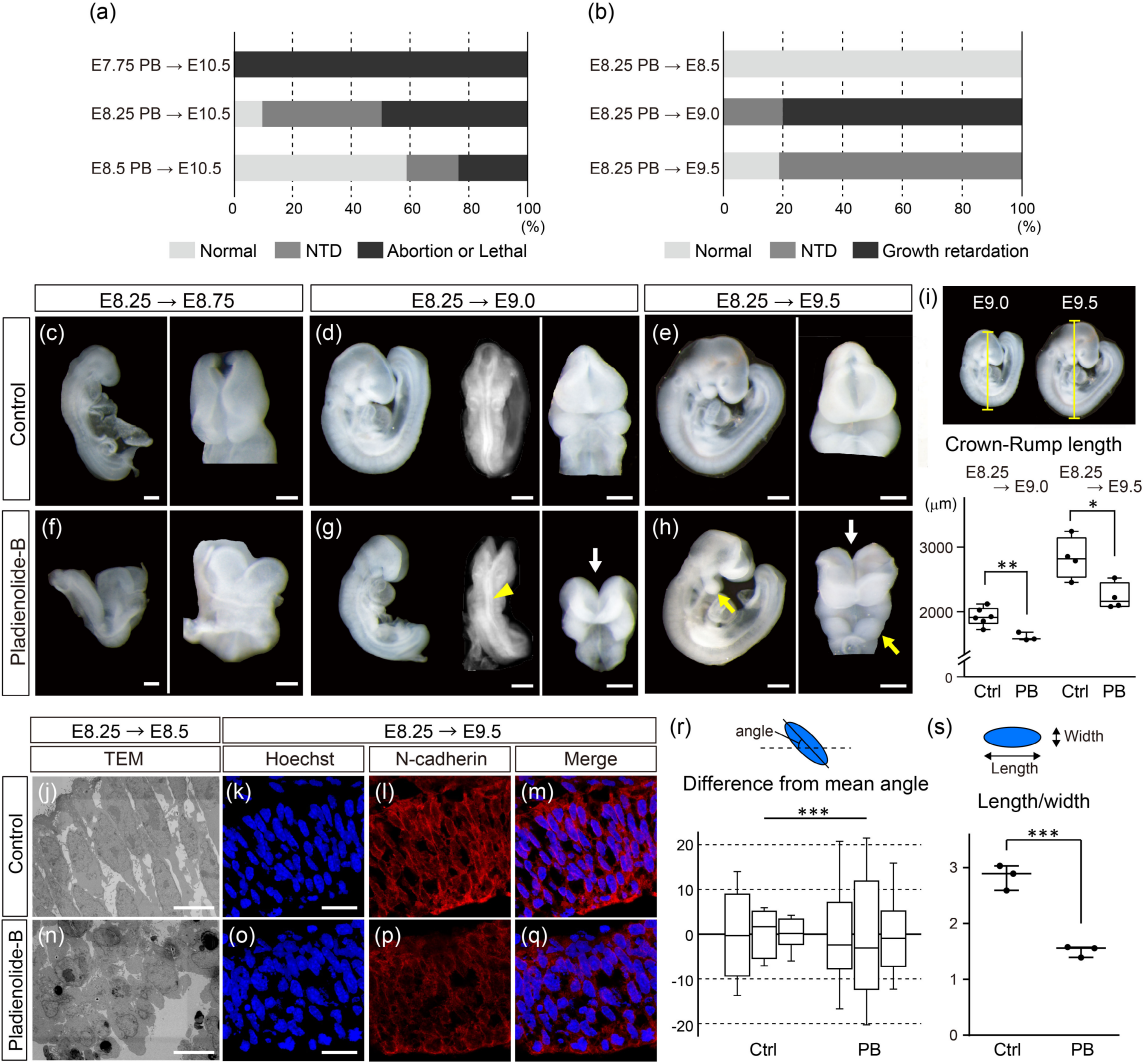
Phenotype of wild‐type embryos exposed to Pladienolide‐B (PB). (a) Ratio of different phenotypes observed in PB‐exposed wild‐type (ICR) embryos at E10.5. Phenotypes of embryos exposed to PB at E7.75 (top, *n* = 22), at E8.25 (middle, *n* = 16), and at E8.5 (bottom, *n* = 17). For all experiments, embryos were obtained from at least two pregnant mice. (b) Ratio of phenotypes in wild‐type (C57BL/6J) embryos exposed to PB at E8.25. Phenotype of PB‐exposed embryos examined at E8.5 (top, *n* = 14), at E9.0 (middle, *n* = 20), and at E9.5 (bottom, *n* = 16). For all experiments, embryos were obtained from at least two pregnant mice. (c–h) Comparison of control embryos (c–e) and embryos exposed to PB at E8.25 (f–h), assessed at E8.75 (c, f), E9.0 (d, g), and E9.5 (e, h). White arrows in (g) and (h) show NTD in the forebrain and hidbrain. Yellow arrowhead in (g) and yellow arrow in (h) indicates the neural tube and the PA1, respectively. (i) Crown‐rump length of control embryos and embryos exposed to PB at E8.25, evaluated at E9.0 (left) and E9.5 (right). The dots represent individual embryos obtained from two pregnant mice. (j, n) Transmission Electron Microscopy (TEM) analysis of the neuroepithelium at E8.5 of control embryos (j) and embryos exposed to PB at E8.25 (n). (k–m, o–q) Immunofluorescence imaging of E9.5 neuroepithelium in control embryos (k–m) and embryos exposed to PB at E8.25 (o–q). Hoechst nuclear (k, o) and N‐cadherin staining (l, p), and merge (m, q) images are shown. For fluorescent staining experiment, *n* = 3 obtained from two control pregnant mice (k–m) or *n* = 4 obtained from three PB‐exposed pregnant mice (o–q). Only one sample was examined for TEM analysis, and consistent results as immunostaining were obtained. (r) An illustration showing the method for measuring the angle of the cell nucleus. The angle of the long axis of the nucleus (blue oval) in the neuroepithelium were measured, and the mean angle for each individual was calculated. The difference between the angle of each cell and the mean angle was measured. (*n* = 3, rectangle bars, obtained from two pregnant mice. 15–20 nuclei were examined in each embryo). (s) The length‐to‐width ratio of the nucleus (blue oval) in the neuroepithelium was calculated. The dots represent individual embryos obtained from two control or two PB‐exposed pregnant mice. *: *p* < 0.05, **: *p* < 0.01, ***p < 0.001 (Student's *t*‐test, *F*‐test). Scale bar: 200 μm for (c, f), 400 μm for (d, e, g, h), 10 μm for (j, n), and 20 μm for (k, o).

To determine which developmental stage exhibits greater sensitivity to PB, we intraperitoneally administered PB to pregnant female ICR mice at E7.75, 8.25, and 8.5, which are critical stages for neural tube closure (NTC) and cranial neural crest cell (CNCC) formation. When examined at E10.5, all embryos dosed at E7.75 were aborted (Figure 1a). Approximately 40% of embryos dosed at E8.25 showed neural tube defect (NTD) at the forebrain and midbrain area (Figure 1a). Embryos exposed to PB at E8.5 exhibited less percentage of NTD and over 50% of the embryos appeared normal when examined at E10.5 (Figure 1a). Based on these results, we found that PB has the most impact on development when administered at E8.25.

We next intraperitoneally administered PB to pregnant female of C57B/L6 mice at E8.25 and examined embryos at E8.5, 8.75, 9.0, and 9.5. All embryos observed at E8.5 had similar appearance to control embryos, probably because body rotation and NTC do not start at this stage (Figure 1b). In contrast, when observed at E8.75 and 9.0, growth retardation was clearly observed, characterized by a delay in body rotation (Figure 1c,f). NTD was also found from the forebrain to hindbrain areas (Figure 1d,e,g,h, white arrow), but the cervical and trunk areas were normally fused (Figure 1g, yellow arrowhead). Notably, over 80% of the embryos at E9.5 showed NTD but they appeared to have the normal first pharyngeal arch (PA1, Figure 1b,e,h, yellow arrow). The body size of embryos was obviously smaller in PB‐administered embryos (Figure 1c–h). The crown‐rump length was significantly shorter in PB‐exposed embryos than control embryos at E9.0 and E9.5 (Figure 1i).

Transmission electron microscope (TEM) observation (Figure 1j,n) and Hoechst staining of histological sections of the neural tube (Figure 1k,o) indicated that the nucleus in the neuroepithelium of the control at E8.5 and E9.5 lined with regularity while PB‐exposed embryos showed fragmented, disturbed arrangement and partly agglutinated nucleus. N‐cadherin, one of the cell adhesion molecules in the neural tube, visualized the cell membrane of the neural tube (Figure 1l,m,p,q). To quantify the arrangement of neuroepithelial cells, the orientation of the longest cell axis was measured (Figure 1r). The difference from the mean angle of each cell exhibited significantly greater variance in PB‐exposed embryos, indicating that the arrangement of the neuroepithelial cells were more irregular in those embryos (Figure 1r). We also measured the length and width of the nuclei to examine cell shape. The length‐to‐width ratio was much larger in the PB‐exposed embryos (Figure 1s). These results demonstrated that the shape of neuroepithelial cells rounded compared to those of their control embryos.

### 3.2 Embryos Exposed to PB Exhibited Exencephaly but Relatively Normal Structure in the Midface and Jaw

**FIGURE 2:**
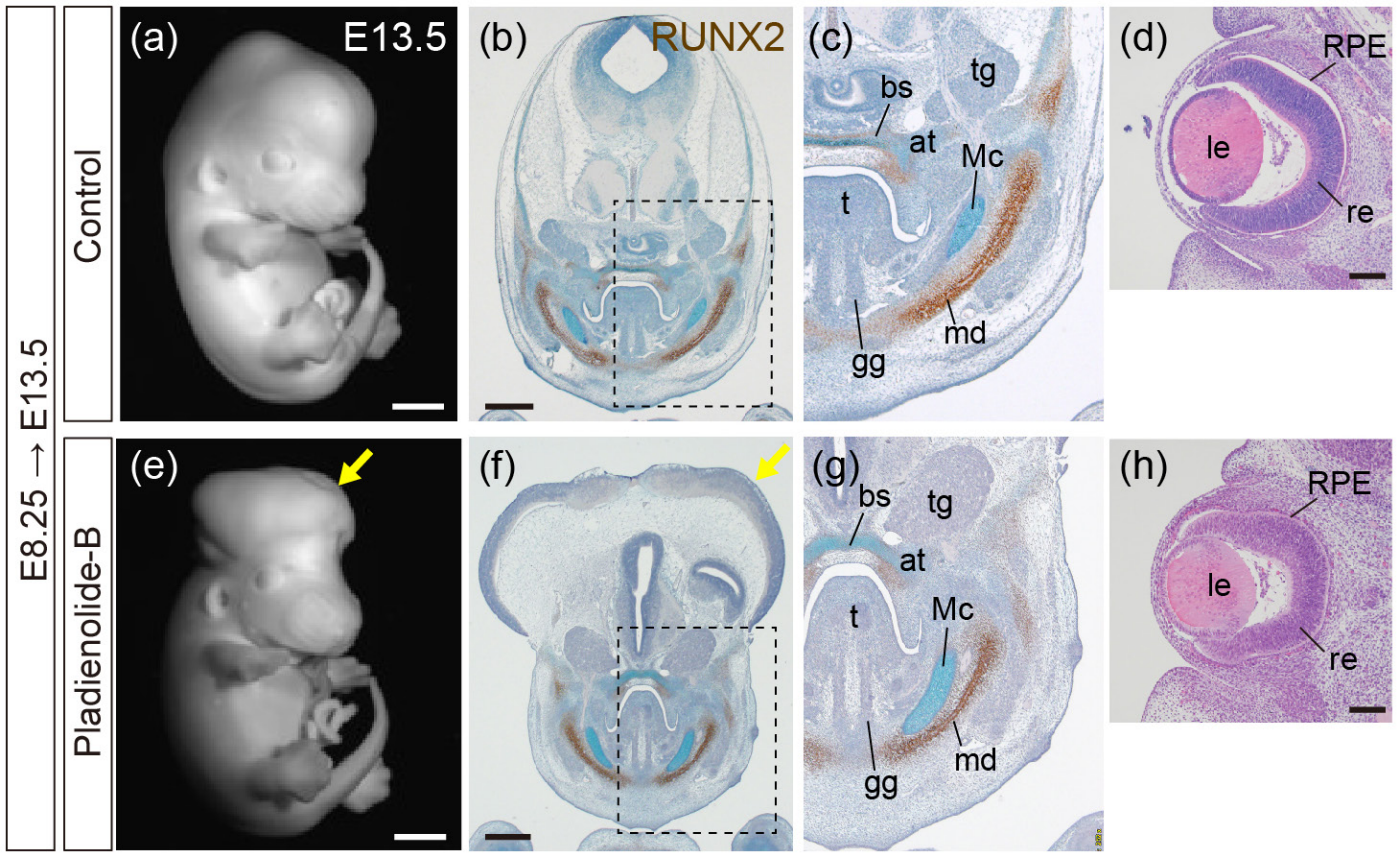
Histological analysis of wild‐type fetuses exposed to PB. (a, e) Whole‐mount fetuses at E13.5, control (a) and PB‐exposed at E8.25 (e). (b, c, f, g) Immunohistochemistry for RUNX2 (brown) with Alcian blue and Toluidine blue staining. Frontal sections of E13.5 control fetuses (b, c) and fetuses exposed to PB at E8.25 (f, g). (c, g) Magnified views of the areas outlined by hatched lines in (b, f). (d, h) Hematoxylin–Eosin staining of frontal sections of the eye at E13.5 in control fetuses (d) and fetuses exposed to PB at E8.25 (h). bs, basisphenoid bone; tg: Trigeminal ganglion; at, ala temporalis; t, tongue; Mc, Meckel cartilage; gg, genioglossus muscle; le, lens; md, mandible; re, retina; RPE, retinal pigment epithelium. Scale bar: 1 mm for (a, e), 500 μm for (b, f), and 100 μm for (d, h). *n* = 3 obtained from one ICR pregnant mouse, and consistent results were obtained.

We next tried to examine craniofacial structures at later stages after administration of PB. However, most of the pregnant females exposed to PB at E8.25 did not sustain pregnancy, and we obtained embryos from only one litter conducted with the ICR strain at E13.5. As expected by NTD at earlier stages (Figure 1), PB‐treated fetuses showed exencephaly (Figure 2a,b,e,f). Histological sections with immunostaining of RUNX2, one of molecular markers of early bone formation, with Alcian blue staining were examined. The trigeminal ganglion, cranial base, Meckel's cartilage, and mandible, most of which are derived from the PA1, were normally developed in PB‐treated fetuses (Figure 2c,g). PB‐exposed fetuses showed a longitudinally elongated shape of the tongue, which is probably due to the pressure exerted on the facial regions from exencephaly (Figure 2c,g). Intrinsic tongue muscles such as the genioglossus were also comparable in PB‐exposed fetus (Figure 2c,g). Since spliceosomopathies includes defects in retina development, the retina was also examined in the fetuses. Although slight deformation probably due to pressure caused by exencephaly was found, the retina was normally developed in PB‐treated fetus (Figure 2d,h). Taken together, the mouse fetus treated with PB exhibited exencephaly but normal development in other craniofacial components including the midface, jaw, and retina.

### 3.3 Apoptosis and Lower Cell Proliferation Were Detected in PB‐Exposed Mouse Embryos

**FIGURE 3:**
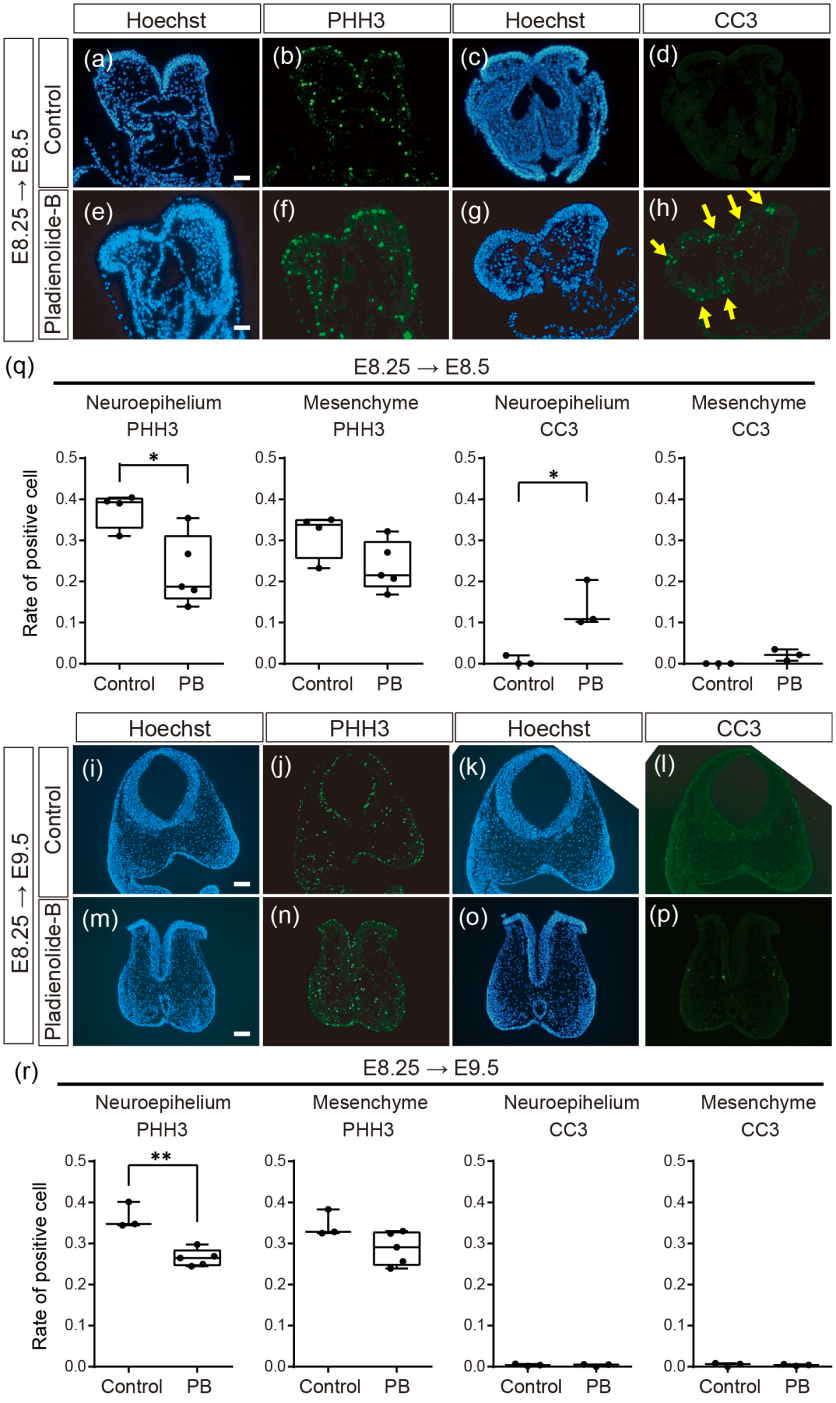
Immunohistochemistry of PHH3 and CC3 in wild‐type embryos. (a–d) Frontal sections of control embryos at E8.5 and (e–h) frontal sections of E8.5 embryos exposed to PB at E8.25. (a, c, e, g) Hoechest, (b, f) PHH3, and (d, h) CC3 immunofluorescent signals. (i–l) Frontal sections of control embryos at E9.5 and (m–p) and frontal sections of E9.5 embryos exposed to PB at E8.25. (i, k, m, o) Hoechest, (j, n) PHH3, and (l, p) CC3 immunofluorescent signals. *n* = 3 for each experiment in (a–h) and (i–p). (q, r) Ratio of PHH3 and CC3 positive cells in the neuroepithelium and mesenchyme in E8.5 (q) or E9.5 (r) embryos of control and PB‐exposed at E8.25. For (q) and (r), the cell rates were calculated by the positive cell number per nucleus number in the same area. The dots in (q) and (r) represent individual embryos obtained from at least two control or two PB‐treated pregnant mice. *: *p* < 0.05, **: *p* < 0.01 (Student's *t*‐test). Scale bar: 50 μm for (a, e) and 100 μm for (i, m).

We examined the ratio of cell proliferation and cell death in the head region of embryos treated with PB at E8.25, examining at E8.5, where no morphological abnormalities were observed (Figure 1b), and E9.5, which showed NTD (Figure 1b). Phospho‐Histone H3 (PHH3), a marker of mitosis, was detected in the neuroepithelium and mesenchyme in the head region including the PA1 (Figure 3b,f,j,n). The rate of the PHH3‐positive cells in the neuroepithelium was significantly lower in the PB‐treated embryos at E8.5 and E9.5, while signals in mesenchyme were not significantly different (Figure 3q,r). Cleaved caspase 3 (CC3), a marker of apoptosis, was also examined to check cell death activity. Although control embryos at E8.5 and E9.5 showed few CC3‐positive cells in either neuroepithelium or mesenchyme at the head region, the neuroepithelium in PB‐exposed embryos exhibited CC3 signals in the large number cells at E8.5 (Figure 3d,h,q, yellow arrows). In contrast, CC3‐positive cells were not detected in PB‐treated embryos at E9.5 (Figure 3l,p,r).

### 3.4 PB‐Exposed *Sf3b4* ^+/−^ Embryos Showed More Severe Phenotype Than Littermate Wild‐Type Embryos

**FIGURE 4:**
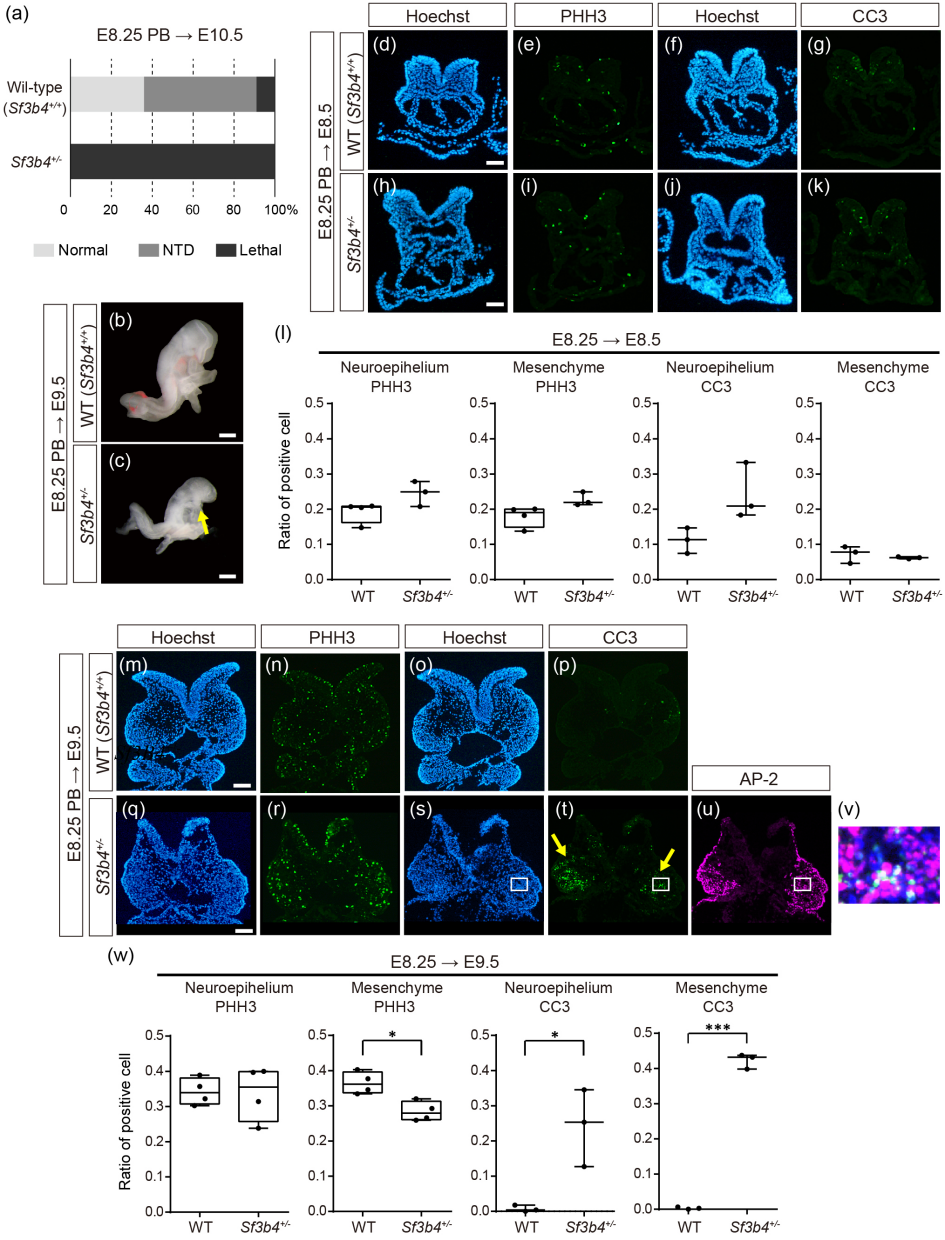
Phenotype of *Sf3B4* ^+/−^ embryos exposed to PB. (a) Ratio of phenotype of wild‐type (top, *n* = 11) and *Sf3B4* ^+/−^ (bottom, *n* = 6) embryos at E10.5 exposed to PB at E8.25. Embryos were obtained from three pregnant mice. (b) Wild‐type and (c) *Sf3B4* ^+/−^ embryos at E9.5 exposed to PB at E8.25. Yellow arrow indicates the PA1 in the *Sf3B4* ^+/−^ embryo. (d–k) Frontal sections of E8.5 wild‐type (d–g) and *Sf3B4* ^+/−^ (h–k) embryos exposed to PB at E8.25. (d, f, h, j) Hoechest, (e, i) PHH3, and (g, k) CC3 fluorescent signals. (l) Ratio of PHH3 and CC3 positive cells in the neuroepithelium and mesenchyme in E8.5 wild‐type and *Sf3B4* ^+/−^ embryos exposed to PB at E8.25. (m–u) Frontal sections of wild‐type (m–p) and *Sf3B4* ^+/−^ (q–u) embryos at E9.5 exposed to PB at E8.25. (m, o, q, s) Hoechest, (n, r) PHH3, and (p, t) CC3 fluorescent signals. (u) Fluorescent signal of AP2 on the same section as (t). (v) Overlay image of the white box area in (s–u). Cells overlapping CC3 (green) and AP2 (magenta) signals are indicated in white. *n* = 3 for each experiment in (d–k) and (m–v). (w) Ratio of PHH3 and CC3 positive cells in the neuroepithelium and mesenchyme in E9.5 wild‐type and *Sf3B4* ^+/−^ embryos exposed to PB at E8.25. For (l) and (w), the cell rates were calculated by the positive cell number per nucleus number in the same area. The dots in (l) and (w) represent individual embryos obtained from at least two pregnant mice. *: *p* < 0.05, ***: *p* < 0.001 (Student's *t*‐test). Scale bar: 400 μm for (b, c), 50 μm for (d, h) and 100 μm for (m, q).

PB exposure at E8.25 to wild‐type mice embryos induced NTD, but CNCCs and craniofacial structures were not affected (Figures 1 and 2). As a further investigation, we exposed PB to *Sf3b4* heterologous knockout embryos. *Sf3b4* ^*+/−*^ mice exhibits smaller body and brain, but they are normal in mating behavior, reproductive ability, and survival rate (Yamada et al. 2020). After mating *Sf3b4* ^*+/−*^ adult females with adult wild‐type (C57B/L6) males, the females were exposed to PB. All *Sf3b4* ^+/−^ embryos exposed at E8.25 were lethal while about 40% of the littermate wild‐type was normal and half of them showed NTD at E10.5 (Figure 4a). Furthermore, the PA1 in mutant embryos was much smaller compared to littermate wild‐type embryos (Figure 4b,c, yellow arrow).

We analyzed cell proliferation and apoptosis of the head region in *Sf3b4* ^+/−^ embryos at E8.5 and E9.5 after PB exposure at E8.25. In *Sf3b4* ^+/−^ embryos collected at E8.5, the rate of PHH3‐ and CC3‐positive cells in both of the neuroepithelium and mesenchyme showed no significant difference compared to littermate wild‐type embryos (Figure 4e,g,i,k,l). In contrast, mutant embryos checked at E9.5 showed significant decrease of cell proliferation rate in the mesenchyme in the midface and PA1(Figure 4n,r,w) and intense signals of cell death in the neuroepithelium and mesenchyme (Figure 4p,t, yellow arrows). To examine the cell type of CC3‐positive cells, immunofluorescent staining of AP2, a marker of the neural crest cell, was conducted to the same histological sections (Figure 4s–u). CC3‐positive signals overlapped with AP2 signals, indicating that apoptosis occurred in CNCCs (Figure 4v). These results demonstrated that PB administration alone does not cause abnormalities in CNCCs, but when additional disturbance of the SF3b complex, downregulation of the expression of *Sf3b4*, apoptosis occurred in CNCCs in mice. Since all PB‐exposed *Sf3b4* ^+/−^ embryos were lethal at E10.5 (Figure 4a), we were unable to conduct morphological analysis of craniofacial structure at later stages.

### 3.5 Morphological Effect of PB Exposure on Zebrafish Embryos at 24 hpf

**FIGURE 5:**
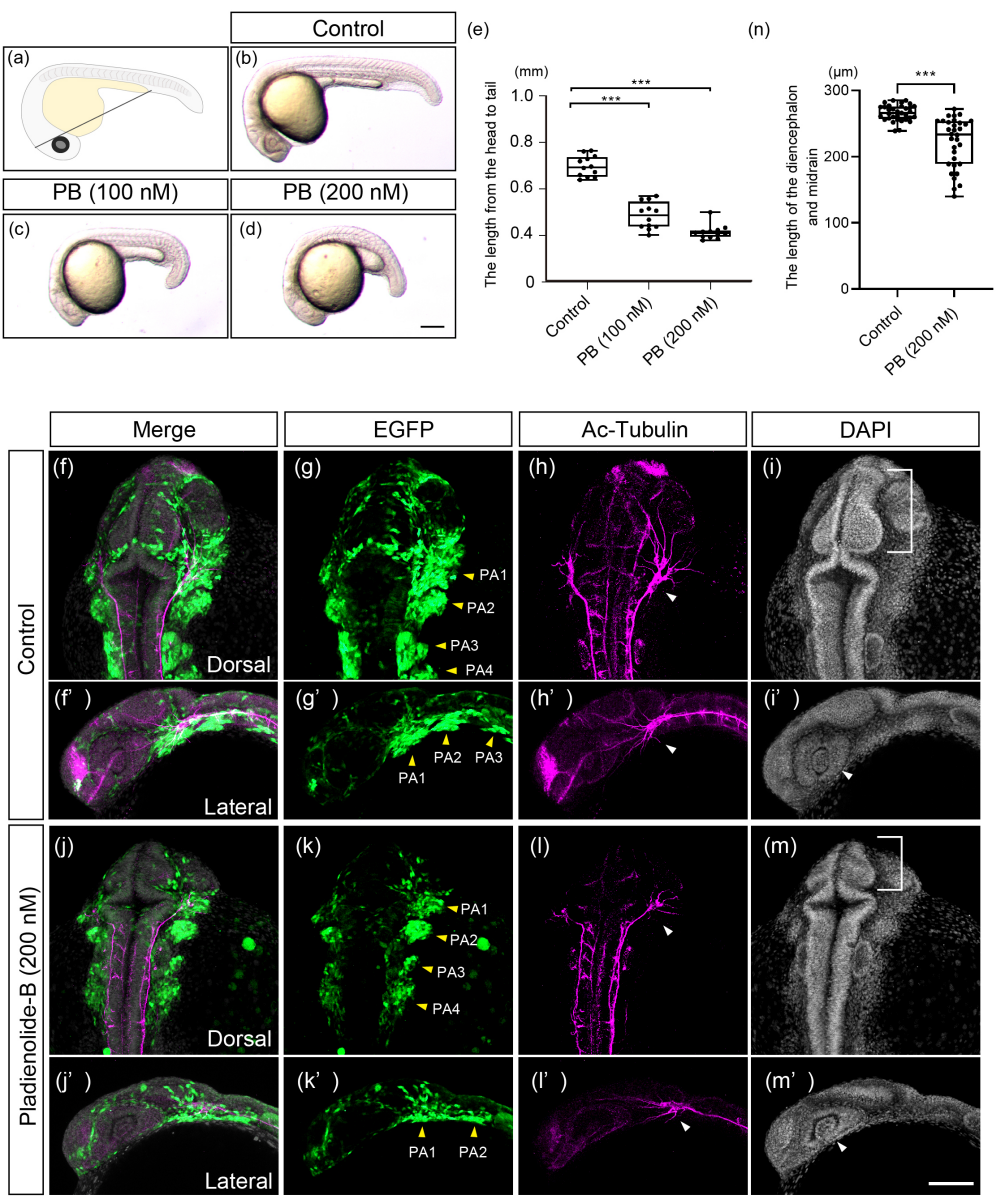
Morphological phenotype resulting from PB treatment in zebrafish embryos at 24 hpf. (a–d) Comparison of overall morphology and body length between control embryos and PB‐treated embryos. Black line in the panel (a) indicates the measured length from the head to tail. (e) Quantification of the length from the head to tail. ***p < 0.001 (one‐way ANOVA followed by Dunnett's multiple comparison test). (f–m′) Effects of PB (200 nM) on neural crest cells in the pharyngeal arch (PA)s which is addressed by the yellow arrowheads (g–k′) and major axonal trajectories and commissure neurons (h–i′). The trigeminal ganglion is marked by the white arrowhead. Brain (midbrain) is indicated by the bracket ((i), (m)), and eye is indicated by the arrowhead ((i′), (m′)) were analyzed. *n* = 30 for experiment for (f–i) and (j–m). (n) Quantification of the length of the midbrain shown in panels (i) and (m). ***: *p* < 0.001 (one‐way ANOVA followed by Dunnett's multiple comparison test). Scale bar: 100 μm. The dots in (e) and (n) represent individual embryos examined. All experiments were performed in three biological replicates.

We next investigated effects of PB during embryonic development in zebrafish. We first examined gross morphological effects of PB, on zebrafish embryos at 24 hpf, when the pharyngeal arches (PAs) are formed. PB at 25, 50, 100, 200, 400, or 500 nM was administered to zebrafish embryos from 4 to 24 hpf. The PB‐exposed embryos at 25 and 50 nM showed no morphological abnormalities and at 400 and 500 nM were lethal. The embryos treated at 100 and 200 nM survived but exhibited morphological defects. The body length of the embryos (the length from the head to tail; corresponding to crown‐rump length in mice, black line in Figure 5a) was shortened in a dose‐dependent manner (Figure 5b–d). The body length was significantly decreased by the PB treatment in a dose‐dependent manner (Figure 5e).

We decided to adopt 200 nM of PB treatment for zebrafish embryos and examined the effect on PAs, brain, and cranial nerves after PB treatment using *sox10:EGFP* in which CNCCs are visualized by EGFP (Figure 5f–m′). The number of PAs (PA1‐4) were not affected; however, malformed morphology of PAs were observed (Figure 5f–g′,j–k′). In particular, morphology of the PA1 was severely affected (Figure 5g,g′,k,k′). Defects in cranial nerves were analyzed by pan‐neural marker, acetylated tubulin (Ac‐Tubulin) staining at 24 hpf. Neuronal defects were observed in PB‐treated embryos (Figure 5h,h′,l,l′). Neuronal projections from the trigeminal ganglion and anterior commissure are evident at this developmental stage and were severely affected by PB treatment (Figure 5h,h′,l,l′ arrowhead, Videos S1 and S2). These embryos also showed smaller brain (midbrain) and eye compared to control embryos (Figure 5i,m bracket, i′, m′ arrowhead). The brain size was significantly decreased by PB treatment (Figure 5n).

### 3.6 PB Treatment Resulted in Structural Abnormalities in the Craniofacial Skeleton, Brain, and Retina in Zebrafish Embryos

**FIGURE 6:**
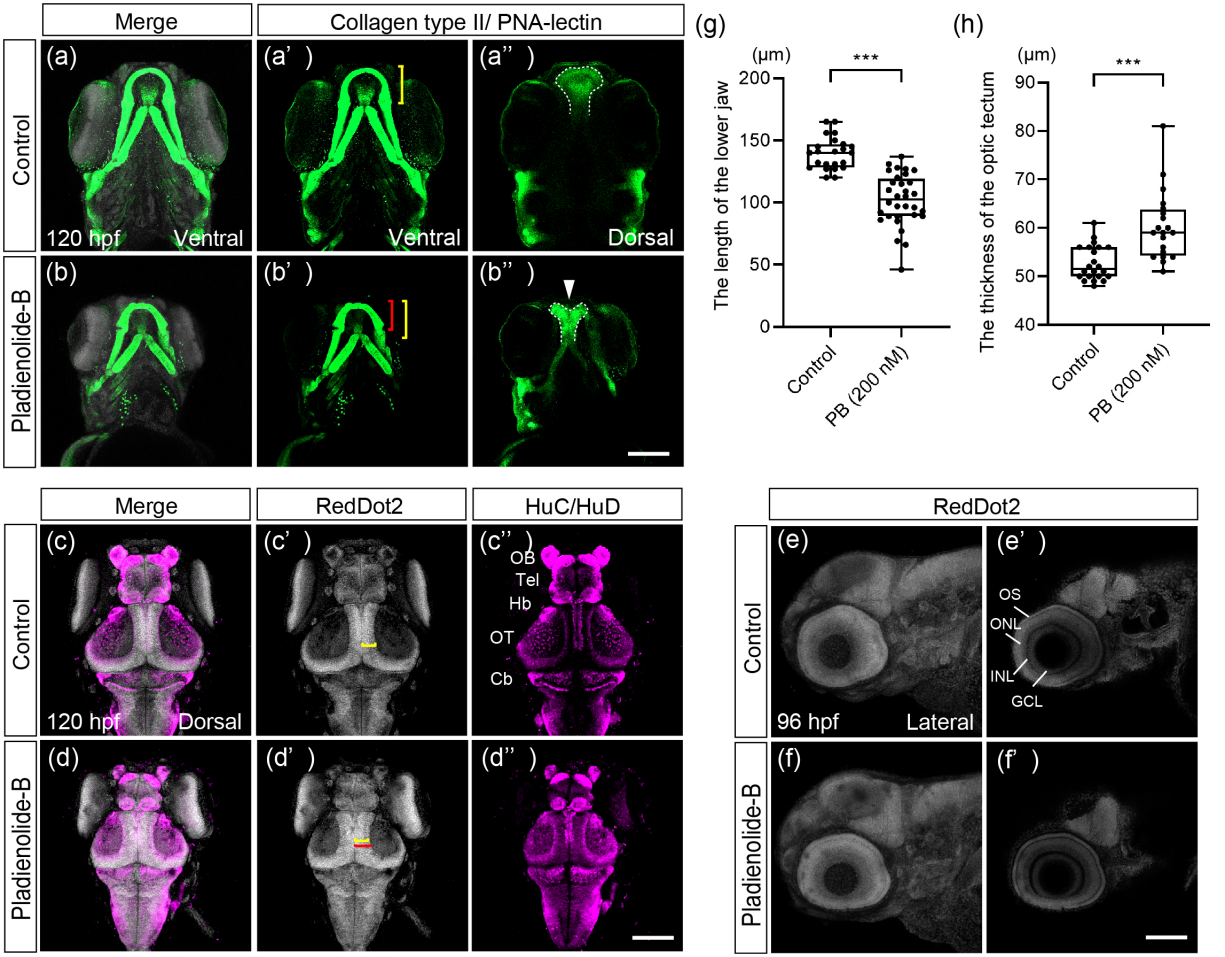
Craniofacial malformation and structural abnormalities of the brain and eye in PB‐exposed embryos at 96 and 120 hpf. (a–b″) Immunofluorescence images of the control and PB‐treated (200 nM) embryos. Samples were stained with anti‐collagen type II antibody and PNA‐lectin to visualize facial cartilages. The Meckel's cartilage ((a′), (b′), ventral view) and ethmoid plate ((a″), (b″), dorsal view) indicated by white arrowhead were analyzed. The bracket indicates the length of the Meckel's cartilage in control (yellow bracket) and PB‐treated embryos (red bracket), while dashed lines outline the ethmoid plate. (c–d″) Samples were stained with anti‐HuC/HuD and RedDot2 to analyze brain defects. Bracket shows the thickness of optic tectum in control (yellow bracket) and PB‐treated embryos (red bracket). (e–f′) Eye morphology ((e), (f)) and eye layer ((e′), (f′)) were analyzed. (g) Quantification of the length of the lower jaw shown in panels (a′) and (b′). (h) Quantification of the thickness of the optic tectum shown in panels (c′) and (d′). ***: *p* < 0.001 (one‐way ANOVA followed by Dunnett's multiple comparison test). Cb, cerebellum; GCL, ganglion cell layer; Hb, habenula; INL, inner nuclear layer; OB, olfactory bulb; ONL, outer nuclear layer; OS, outer segment of photoreceptor; OT, optic tectum; Tel, telencephalon. Scale bars: 100 μm. *n* = 19, 28, 21, and 28 for experiment for (a, c), (b, d), (e), and (f), respectively. The dots in (g) and (h) represent individual embryos examined. All experiments were performed in three biological replicates.

We next analyzed craniofacial abnormalities, brain malformation, and retinal defects at later stages. PB‐treated embryos displayed severe defects in PA1 morphology, and these individuals showed micrognathia and cleft palate (Figure 6a–b″, g). Defects in the brain were analyzed by nuclear stating for brain morphology and anti‐HuC/HuD staining for neurons. PB‐treated embryos exhibited smaller brain region; however, thickness in the optic tectum was significantly increased (Figure 6c–d″, h). As for eye and retinal development, the size of lens and the number of retinal layers were not affected; however, each retinal layer was shortened (Figure 6e–f′).

### 3.7 Cell Activities and Migration of CNCCs in the PA1 Affected in PB‐Exposed Zebrafish Embryos

**FIGURE 7:**
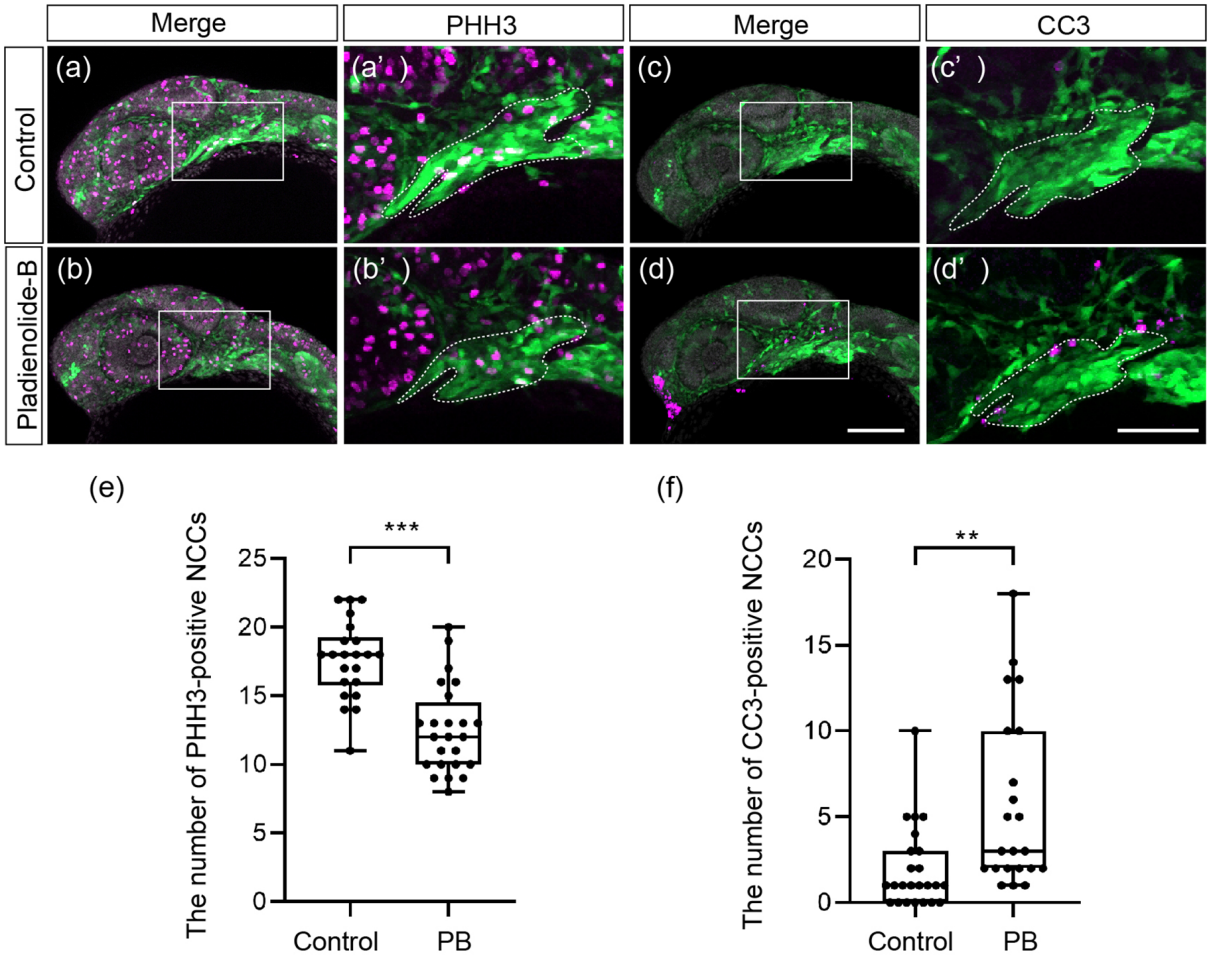
Mitosis and apoptosis in migratory CNCCs and CNCCs in PB‐treated embryos at 24 hpf. (a–b′) Immunofluorescence images of mitotic ability of CNCCs. Samples were stained with anti‐GFP antibody and anti‐PHH3 antibody. (a′, b′) Magnified view of the rectangle in the panels (a) and (b). Green and magenta represent represents CNCCs and mitotic cells, respectively. The PA1 is defined by dashed line. (c–d′) Immunofluorescence images of apoptosis in CNCCs. Samples were stained with anti‐GFP antibody and anti‐CC3 antibody. (c′, d′) Magnified view of the rectangle in the panels (c) and (d). Green and magenta represent CNCCs and apoptotic CNNCs, respectively. *n* = 22 (control), *n* = 22 (PB 200 nM). (e) Quantification of the number of PHH3‐positive migratory CNCCs and CNCCs in the PA1. (f) Quantification of the number of CC3‐positive migratory CNCCs and CNCCs in the PA1. ***: *p* < 0.001 (one‐way ANOVA followed by Dunnett's multiple comparison test). Scale bars: 100 μm (a–d), 50 μm (a′–d′). The dots in (e) and (f) represent individual embryos examined. All experiments were performed in three biological replicates.

Since obvious developmental abnormality was detected by observation of EGFP‐positive CNCCs in *sox10:EGFP* zebrafish, we next investigated the PA1 morphology and CNCC dynamics after PB treatment. We examined cell proliferation and apoptosis in premigratory and migratory CNCCs during the first 24 h of development. Cell proliferation and cell death of CNCCs in the PA1 were analyzed by immunofluorescence staining using PHH3 and CC3 antibodies (Figure 7a–d′). The number of the PHH3‐positive CNCCs significantly decreased in PB‐treated embryos (Figure 7e). The number of the CC3‐positive CNCCs showed a significant increase in PB‐exposed embryos (Figure 7f). Collectively, these findings suggest that decreased mitotic ability and increased apoptosis in CNCCs could contribute to the PA1 dysplasia, ultimately leading to the craniofacial malformations in the later stages.

**FIGURE 8:**
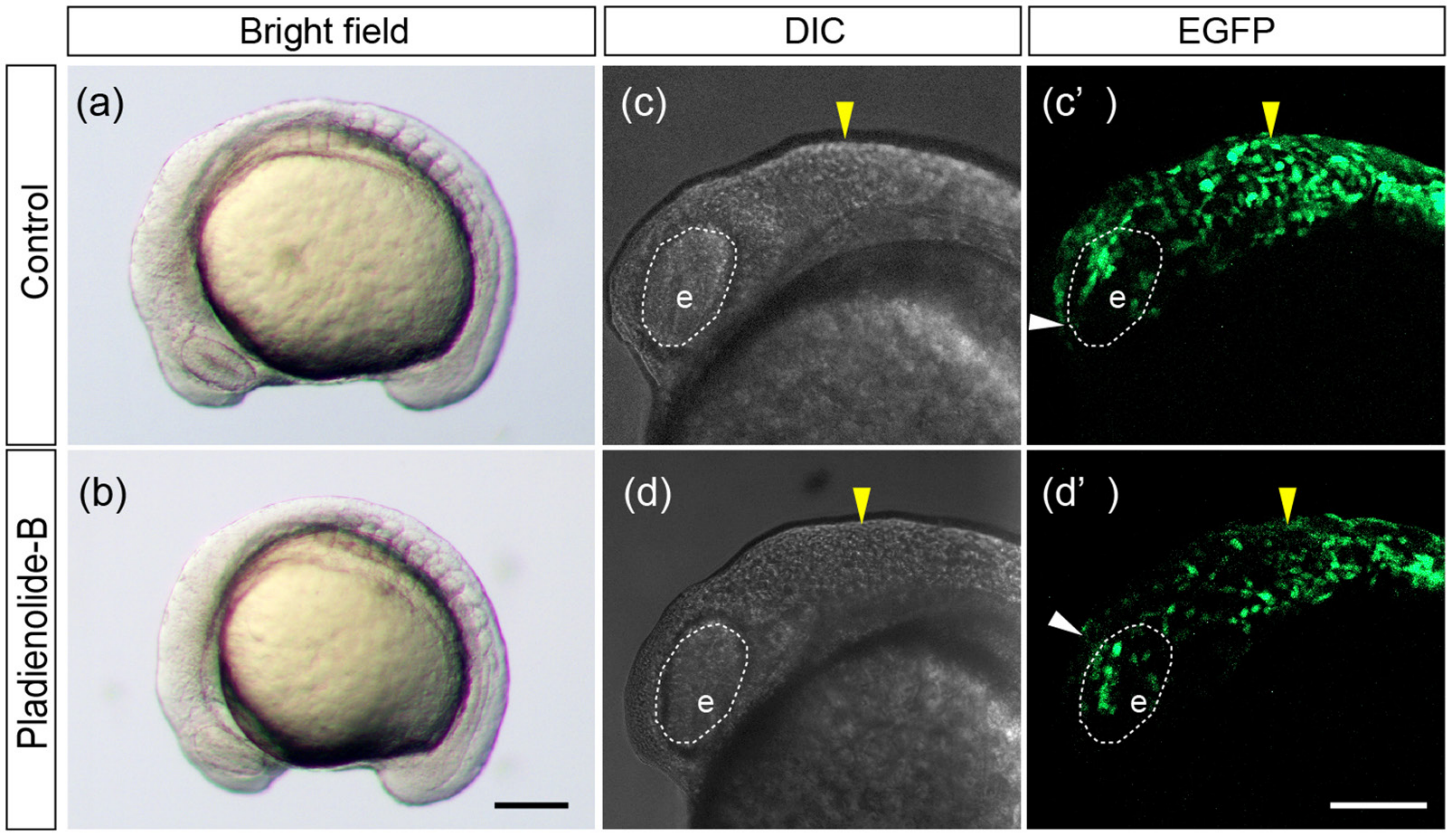
CNCCs migration in PB (200 nM)‐treated *sox10:EGFP* embryos at 13 ss. (a–b) Overall defects were analyzed at 13 ss. (c–d′) Live DIC (differential interference contrast, (c), (d)) and fluorescence imaging (c′, d′) of CNCC migration in control and PB‐treated embryos. The yellow arrowheads in (c) and (d) indicate the position of the midbrain‐hindbrain boundary (MHB) at which CNCCs start migration. The white arrowhead in (c′) and (d′) indicates the frontier of migratory CNCCs via the frontonasal pathway. Dashed line indicates the eye (e). Scale bars: 100 μm. *n* = 30 for each experiment. All experiments were performed in three biological replicates.

We next investigated effect on CNCC migration after PB treatment. CNCCs typically migrates from the midbrain and anterior hindbrain region at 10 ss to reach the dorsal side of the eye along the frontonasal pathway (Liu et al. 2023). We examined CNCC migration at the 13 ss when the number of somites remained unaffected as evidenced by observations of typical somite formation in *sox10:EGFP* embryos (Figure 8a,b). This indicates that the overall developmental progression, specifically somite segmentation giving rise to various tissues and organs during embryogenesis, was not disrupted by the treatment. The frontier of migratory CNCCs via the frontonasal pathway is at the dorsal side of the eye at the 13 ss (Figure 8c,c′,d,d′ white arrowhead). However, a notable migration defect in CNCCs was observed in PB‐treated embryos (Figure 8c′,d′, white arrowhead). Additionally, PB treatment led to a decrease in migratory CNCCs (Figure 8c′,d′). These results suggest that PB inhibits the migration of CNCCs and their survival potentially giving rise to PA1 malformation.

## 4 Discussion

In this study, we successfully mimicked the phenotypes of spliceosomopathies in mice partially and in zebrafish by administering PB to embryos. PB has been demonstrated in multiple in vitro studies to affect splicing patterns (Teng et al. 2017; Finci et al. 2018; Larsen 2021). Additionally, recent in vivo knockout analyses have revealed that inhibition of a component of the SF3b complex impacts splicing patterns and gene expression (Ulhaq et al. 2023a, 2023b; Ulhaq and Tse 2024; Griffin et al. 2024). Based on these findings, the zebrafish administered PB in the present study could serve as a novel model for researching the function of the SF3b complex and the causes of spliceosomopathies.

In PB‐exposed mouse embryos, growth retardation occurred, and the body size was decreased (Figure 1). These results are consistent with previous studies that patients in spliceosomopaties exhibit short stature (Lehalle et al. 2015). Cell proliferation rate also decreased, and cell death rate increased in the head neuroepithelium (Figure 3), suggesting that inhibition of the function of the SF3b complex sensitively affects the early stage of brain formation in mice. NTC requires dynamic changes in the cell shape of the neural plate (Nikolopoulou et al. 2017). Given that most cells in the head neuroepithelium of PB‐treated embryos showed spherical deformation (Figure 1), such cell morphological changes may inhibit NTC. Further studies are needed to reveal how NTD occurs by PB administration.

Craniofacial malformations are generally caused by defects in the development of CNCCs (Vega‐Lopez et al. 2018). Therefore, we targeted the developmental stages of CNCCs for administration of PB in the mouse. While we initially hypothesized that inhibiting the function of the SF3b complex would affect CNCCs in the mouse embryo, no abnormalities were detected in their formation or development of the midface and jaws (Figures 1 and 2). However, these finding corroborate the results in *Sf3b4* ^*+/−*^ mice, where brain formation is affected but CNCCs remain unaffected (Yamada et al. 2020). Interestingly, a reduction in the expression level of *Sf3b4*, combined with PB exposure, influenced the development of both the brain and CNCCs (Figure 4), suggesting that CNCCs are comparatively less sensitive to disruption of the SF3b complex than the brain in craniofacial development. Previous studies propose that various cell types forming the craniofacial structure (endoderm, mesoderm, ectoderm, and CNCCs) exhibit distinct requirements for efficient splicing (Beauchamp et al. 2020). Consequently, we expect that *Sf3b4* ^*+/−*^ mice, including a certain splicing abnormality in systemic cells, show cell death only in the neuroepithelium (Figure 1), but PB exposure to *Sf3b4* ^*+/−*^ mice impose additional burden of splicing disruption, causing them to fall below the splicing efficiency threshold in CNCCs (Figure 4). Less is known about why each cell type demonstrates different splicing thresholds for normal development, which should be investigated in future studies (Olthof, White, and Kanadia 2022). It is also noteworthy that although previous knockout analyses of a SF3b component have not shown any significant impact on CNCCs in mice, the present study first provides evidence that components in the SF3b complex plays a crucial role in development of CNCCs in mice. However, PB‐exposed *Sf3b4* ^*+/−*^ embryos exhibited lethal by E10.5 (Figure 4), and we were unable to examine craniofacial defects in later stages. These results may indicate the limitations of studies involving the administration of splicing inhibitors to mice.

In contrast to mice, zebrafish displayed prolonged survival even following exposure to PB, allowing for comprehensive analysis of morphological abnormalities. Similar to the observations in mice, a significant reduction in body size was evident (Figure 5). The development of the brain, cranial nerve, retina, palate, and jaw were affected (Figure 6). Additionally, a decrease in the cell proliferation rate and an increase in cell death within CNCCs were observed (Figure 7). These findings align with previous studies demonstrating the influence of *sf3b1* mutations on brain, cranial nerve, and neural crest formation in zebrafish (An and Henion 2012), as well as the consequences of *sf3b4* homologous knockout in zebrafish on retinal and craniofacial formation (Ulhaq et al. 2023b; Ulhaq and Tse 2024). Furthermore, many of the identified phenotypes in spliceosomopathies were concurrently identified in PB‐exposed zebrafish, likely due to the administration of the highest concentration of PB that did not result in embryonic lethality (200 nM). Interestingly, despite PB exposure disrupting brain development in zebrafish, NTD resembling those in mice were not observed (Figure 5). This difference may be attributed to distinct mechanisms of NTC between mice and zebrafish (Araya et al. 2016).

Based on the previous knockout and morpholino analyses in model organisms, it is evident that heterozygous deletion of a SF3b component has a minor impact on morphogenesis but exerts a significant effect at lower expression levels (An and Henion 2012; Isono et al. 2005; Yamada et al. 2020; Kumar et al. 2023; Griffin et al. 2024). Notably, homozygous deletion of a SF3b component allows survival until a specific developmental stage in zebrafish and *Xenopus*; however, in mice, it results in early embryonic lethality (Ulhaq et al. 2023b; Yamada et al. 2020; Griffin et al. 2024). Based on these data, we posit that the low lethality threshold in mice contributes to many embryonic deaths in PB‐exposed mice in this study (Figure 1). The divergence in outcomes among model organisms may be associated with the distinct timing of the maternal to zygotic transition in these species, but further studies are required to unveil underlying mechanisms (Olthof, White, and Kanadia 2022).

It is also crucial to consider that administered PB in the mother should undergo metabolism in mice. PB‐treated mouse embryos illustrated a downregulation of cell proliferation and an increase in cell death in the neuroepithelium within six hours of PB administration (Figure 3). However, after approximately a day of PB administration, cell death is no longer evident in the neuroepithelium (Figure 3), likely due to PB metabolism in the mother and the subsequent dissipation of its effects within the given timeframe. In addition, continuous administration to pregnant mice is found to be difficult in mice. We also tested another splicing inhibitor, Herboxidiene, but obtaining data were challenging due to a sharp increase in lethality with increasing doses compared to PB (data not shown). Given these difficulties, it is plausible that our study only captured abnormal brain formation as one of the most sensitive outcomes of PB administration in mice.

On the contrary, in zebrafish, we achieved a stable suppression of SF3b function through the utilization of our previously established chemical exposure method (Liu et al. 2020, 2023; Narumi et al. 2020). This approach facilitated the establishment of a model that consistently suppresses SF3b function, effectively mimicking the phenotypes observed in genetic modification analyses and in human patients. In this study, we focused on CNCCs, revealing that the SF3b complex is implicated not only in proliferation and survival but also in the migration of CNCCs in zebrafish. Taking advantage of the accessibility of zebrafish embryos, we aim to conduct a detailed analysis of abnormalities in CNCCs and other organs in future studies.

In the present study, we found that PB administration in *Sf3b4* ^*+/−*^ mouse embryos resulted in additional phenotypic effects compared to wild‐type embryos (Figure 4), suggesting that the range of phenotypes obtained expands as the impact on the SF3b complex increases. Moreover, considering the proposed “competitive model” of splicing inhibitors, where they competitively interact with pre‐mRNA in the branch point sequence (Teng et al. 2017; Finci et al. 2018; Larsen 2021), we underscore the potential for seamless modulating the impact on the SF3b complex by altering the dose of splicing inhibitors. This modulation may lead to variations in splicing abnormalities, thus influencing the observed phenotypes. We are currently initiating analysis on the changes in phenotypes and splicing patterns influenced by varying concentrations of PB administration in zebrafish. Furthermore, splicing inhibitors also may unveil the time‐dependent function of the SF3b complex by controlling the timing of administration. Although experiments involving the administration of splicing inhibitors to embryos may elucidate the relationship between impaired splicing function and the resulting phenotypes, they cannot directly reveal the mechanisms underlying the development of spliceosomopathies in humans. Despite these limitations, this experimental system is likely to provide valuable insights into the basic mechanisms of splicing and craniofacial development, as well as contribute to understanding the causes of human diseases.

## Conflicts of Interest

S.L., Y.N., M.M., and J.T. are employed by Kao Corporation.

## Supporting information


**Video S1.** Cranial nerves visualized by acetylated tubulin in the control zebrafish embryo at 24 hpf.

**Video S2.** Cranial nerves visualized by acetylated tubulin in the PB‐treated zebrafish embryo at 24 hpf.

## Data Availability

The data that support the findings of this study are available from the corresponding author upon reasonable request.
